# TREM2 Is Associated with Advanced Stages and Inferior Prognosis in Oral Squamous Cell Carcinoma

**DOI:** 10.3390/cancers14194635

**Published:** 2022-09-24

**Authors:** Ann-Kristin Struckmeier, Anne Radermacher, Michael Fehrenz, Dalia Alansary, Philipp Wartenberg, Mathias Wagner, Anja Scheller, Jochen Hess, Julius Moratin, Christian Freudlsperger, Jürgen Hoffmann, Lorenz Thurner, Klaus Roemer, Kolja Freier, Dominik Horn

**Affiliations:** 1 Department of Oral and Maxillofacial Surgery, Saarland University Medical Center, 66421 Homburg, Germany; 2 Institute of Biophysics, Center for Integrative Physiology and Molecular Medicine (CIPMM), Saarland University, 66421 Homburg, Germany; 3 Department of Experimental and Clinical Pharmacology and Toxicology, Center for Molecular Signaling (PZMS), Saarland University, 66421 Homburg, Germany; 4 Department of Pathology, Saarland University Medical Center, 66421 Homburg, Germany; 5 Department of Molecular Physiology, Center for Integrative Physiology and Molecular Medicine (CIPMM), Saarland University, 66421 Homburg, Germany; 6 Department of Otorhinolaryngology, Head and Neck Surgery, University Hospital Heidelberg, German Cancer Research Center (DKFZ), 69120 Heidelberg, Germany; 7 Department of Oral and Maxillofacial Surgery, University Hospital Heidelberg, 69120 Heidelberg, Germany; 8 Department of Internal Medicine 1 (Oncology, Hematology, Clinical Immunology, and Rheumatology), Saarland University Medical Center, 66421 Homburg, Germany; 9 José Carreras Center for Immuno and Gene Therapy, Saarland University, 66421 Homburg, Germany

**Keywords:** TREM2, oral squamous cell carcinoma (OSCC), tumor-associated macrophages (TAMs), tumor microenvironment (TME), sTREM2, immunotherapy

## Abstract

**Simple Summary:**

Triggering receptor expressed on myeloid cells 2 (TREM2) is an appealing candidate for immunotherapy. However, results regarding its role in multiple cancers are rare or even contradictory. We showed that TREM2 expression in immune cells, among which were macrophages in peripheral blood mononuclear cells and tumor-associated macrophages, was associated with advanced tumor stages and reduced survival rates in oral squamous cell carcinoma (OSCC) patients. Our findings implicate that TREM2 could serve as an effective target for prognostic and therapeutic approaches in OSCC patients. Anti-TREM2 immunotherapy might greatly improve patients’ prognosis. High levels of sTREM2 were associated with advanced UICC stages, indicating that sTREM2 might be a biomarker in OSCC patients.

**Abstract:**

Triggering receptor expressed on myeloid cells 2 (TREM2) is suggested to hamper antitumor immune response in multiple cancers. However, the role of TREM2 in oral squamous cell carcinoma (OSCC) and its expression in tumor-associated macrophages (TAMs) are unknown. In this study, TREM2 expression was analyzed in the primary tumors and corresponding lymph-node metastases of OSCC patients via immunohistochemistry on tissue microarrays. Human peripheral blood mononuclear cells (PBMCs) and single-cell suspensions of tumor and healthy adjacent tissues were analyzed for the presence of TREM2^+^ macrophages and TAMs using flow cytometry. The serum levels of soluble TREM2 (sTREM2) were quantified using an enzyme-linked immunosorbent assay. High TREM2 expression was associated with advanced UICC stages (Spearman’s rank correlation (SRC), *p* = 0.04) and significantly reduced survival rates in primary tumors (multivariate Cox regression, progression-free survival: hazard ratio (HR) of 2.548, 95% confidence interval (CI) of 1.089–5.964, *p* = 0.028; overall survival: HR of 2.17, 95% CI of 1.021–4.613, *p* = 0.044). TREM2 expression was significantly increased in the PBMCs of OSCC patients in UICC stage IV compared with healthy controls (ANOVA, *p* < 0.05). The serum levels of sTREM2 were higher in advanced UICC stages, but they narrowly missed significance (SRC, *p* = 0.059). We demonstrated that TREM2 was multi-factorially associated with advanced stages and inferior prognosis in OSCC patients and that it could serve as a prognostic biomarker in OSCC patients. Targeting TREM2 has the potential to reshape the local and systemic immune landscape for the potential enhancement of patients’ prognosis.

## 1. Introduction

Oral squamous cell carcinoma (OSCC) represents the most common malignant neoplasm of the head and neck region. The annual incidence is estimated at 350,000 cases worldwide [1,2].

The 5-year overall survival rate of OSCC patients has remained at approximately 50% over the last decades, despite improvements in treatment approaches, including surgical resection, radiotherapy, and chemotherapy [3]. Especially, patients with locally advanced or metastatic OSCC have a poor prognosis. The successful and individualized therapy of high-stage OSCC remains the major clinical challenge for clinicians.

Cancer immunotherapy has shown remarkable anti-tumor activity in the treatment of recurrent platinum-refractory or metastasized head and neck squamous cell carcinoma (HNSCC) [4]. For instance, immune checkpoint inhibitors (ICIs) pembrolizumab and nivolumab are antibodies that target the programmed cell death-1 (PD-1) receptor. Immune checkpoint receptors, such as PD-1, play crucial roles in evading immune surveillance and dampening antitumor immune responses, especially in the tumor microenvironment. Although ICIs hold great promise, only a small fraction of cancer patients achieve a lasting response to monotherapy, and almost all patients eventually progress to drug resistance [5]. This is attributed to the intricate tumor microenvironment (TME) and immune escape mechanisms of cancer cells [6,7].

Recent research by Molgora et al. provided evidence that triggering receptor expressed on myeloid cells 2 (TREM2) is a potential target in cancer therapy [8]. TREM2 is a transmembrane receptor of the immunoglobulin superfamily. It is implicated in tissue repair [9], neurogenerative disorders [10], obesity [11], atherosclerosis [12], and fatty liver disease [13]. However, studies investigating the role of TREM2 in malignancy are rare, and the results regarding different malignancies are contradictory. Katzenelenbogen et al. demonstrated in a mouse model that TREM2 is a key phenotypic marker for tumor-associated macrophages (TAMs) in the TME [14]. TAMs are suggested to hamper immune response, thereby facilitating tumor proliferation, tumor maintenance, angiogenesis, and metastasis [15,16,17]. They typically exhibit the M2 macrophage phenotype (i.e., anti-inflammatory, pro-tumoral), which can be identified using generic macrophage marker CD68 plus CD163 or CD206 [18].

TREM2 also exists in a soluble form (sTREM2), which is generated through receptor shedding or alternative splicing [19]. sTREM2 can be detected in blood and cerebrospinal fluid and is significantly associated with disease severity in neurological diseases, e.g., multiple sclerosis [20] and Alzheimer’s disease [21]. Despite the critical role of sTREM2 in neurologic disease, its role as a biomarker in malignancies remains poorly understood.

The aim of this study was to investigate and analyze the role of TREM2 in primary tumors and lymph-node metastases (LNMs) in OSCC in order to explore its significance as a potential target for prognostic and therapeutic approaches. Secondly, we examined TREM2 expression in OSCC tumor tissues, healthy adjacent tissues and peripheral blood mononuclear cells (PBMCs) to reveal its link to macrophages. Furthermore, we investigated the levels of sTREM2 in the serum of OSCC patients to evaluate its significance as a potential biomarker.

## 2. Materials and Methods

### 2.1. Patients

Immunohistochemistry (IHC) was performed on tissue microarrays (TMAs) to determine TREM2 expression in primary tumors and LNMs. OSCC patients received diagnosis and primary surgical treatment, including tumor resection and neck dissection, at Department of Oral and Maxillofacial Surgery at University Hospital Heidelberg according to the national guideline for OSCC therapy between 2010 and 2016.

The prospective cohort for flow cytometric analyses and enzyme-linked immunosorbent assays (ELISAs) was recruited at Department of Oral and Maxillofacial Surgery at Saarland University Medical Center between 2020 and 2022. Blood samples, tumor, and healthy adjacent tissues were collected at the time of primary surgical treatment, including tumor resection and neck dissection. Moreover, the blood samples of healthy controls (HCs) were collected. None of the patients enrolled had received previous chemotherapy or radiotherapy, nor did they have a history of prior malignancies. TREM2 expression might be influenced by several diseases; thereby, patients with dementia, depression, diabetes mellitus, and autoimmune diseases were excluded from the HC group.

Written informed consent was obtained from all patients before their involvement in the study. The study design and methods were approved by the ethics committees of Heidelberg University (ethics vote: S-360/2011) and University of Saarland (ethics vote: 37/20).

The histopathological diagnosis and the differentiation grade of the tumors were provided by Department of Pathology at University Hospital Heidelberg and Department of Pathology at Saarland University Medical Center. Tumors were classified according to the currently valid TNM classifications (7th edition for tissue samples stained immunohistochemically and 8th edition for tissue and blood samples for flow cytometry and ELISA).

### 2.2. Immunohistochemistry

TREM2 expression in 171 primary tumors and 33 corresponding LNMs was investigated via IHC. TMAs were prepared following published protocols [22,23].

Before staining, paraffin-embedded TMAs were deparaffinized in xylene (3× for 5 min) and soaked in reducing concentrations of ethanol (2 × 99,8%, 1 × 96%, and 1 × 70%, each for 5 min) to rehydrate. In the next step, the TMAs were boiled in citric acid buffer (pH 6.0) for 30 min to retrieve antigens and cooled down for 20 min at room temperature (RT). TMAs were washed in PBS for 5 min twice. Then, the TMAs were incubated for 10 min with BLOXALL Endogenous Enzyme Blocking Solution (Vector Laboratories, Newark, CA, USA) to suppress endogenous peroxidase activity. TMAs were blocked in 2,5% horse serum (Vector Laboratories) for 1 h and incubated in the 100-fold diluted anti-TREM2 monoclonal antibody (clone D8I4C; Cell Signaling Technology, Cambridge, UK) at 4 °C overnight, rinsed with TBST thrice the next day, and then incubated with the secondary antibody (ImmPRESS Universal Antibody; anti-mouse IgG-anti-rabbit IgG; peroxidase; Vector Laboratories) at RT for 30 min. The TMAs were developed in DAB solution (ImmPACT DAB EpV Substrate; Vector Laboratories). Finally, Mayer’s hemalum solution (1:4 dilution; Merck KGaA, Darmstadt, Germany) was used for counterstaining.

Slides were scanned with Axio Scan.Z1 (Zeiss, Oberkochen, Germany) for further investigation. The TMAs were scored using the ZEN (blue edition) program (Zeiss) by three independent observers.

TMAs were assessed by determining the density of TREM2^+^ immune cells (low = 0–3 cells, high > 4 cells) in representative areas and distinguishing the distribution of TREM2^+^ immune cells between peritumoral and intratumoral regions.

We used a dataset previously published by Moratin et al. [23] to evaluate the correlation between TREM2 and PD-L1/2. In situ hybridization for human papillomavirus (HPV)-DNA was performed following published protocols [24,25]. Immunohistochemical staining targeting p16 was performed following the manufacturer’s instructions (clone EPR1473; Abcam, Cambridge, UK).

### 2.3. Blood Samples

Venous blood was obtained from 19 tumor patients and 4 volunteers for HCs. The serum was clotted for 10 min at RT prior to centrifugation at 1500× g for 5 min. The serum was collected into tubes and placed in Mr. Frosty (Nalgene, Rochester, NY, USA) overnight at −80 °C. The next day, tubes were transferred to nitrogen.

### 2.4. Peripheral Blood Mononuclear Cell Preparation

PBMCs were isolated using centrifugation using a Ficoll density gradient. First, blood samples were diluted at 1:1 with PBS (Thermo Fisher Scientific, Waltham, MA, USA) and thoroughly mixed. Next, 20 mL of Ficoll-Paque medium (Ficoll-Paque Plus; GE Healthcare, Chicago, IL, USA) was poured at the bottom of a 50 mL conical tube, and the cell suspension was carefully layered on top. Tubes were centrifuged at 1750× g for 20 min at RT without applying any brake. The layer of mononuclear cells was transferred to a 50 mL conical tube. Cells were washed twice using 10 mL of PBS each time. The amount and the viability of the cells were assessed with Luna-FX7 Automated Cell Counter (Logos Biosystems, Anyang-si, Korea). Subsequently, cells (2 × 10^6^ cells per tube) were resuspended in 90% FBS and 10% DMSO. Tubes were placed in Mr. Frosty (Nalgene) overnight at −80 °C. The next day, tubes were transferred to nitrogen.

For thawing, cells were placed in a 37 °C water bath and then transferred to pre-warmed RPMI (20 mL). Cells were washed twice using RPMI.

### 2.5. Preparation of Single-Cell Suspension

We obtained fresh tumor samples from 9 patients with primary OSCC. Moreover, we obtained healthy adjacent tissues from 6 of these patients. Tissue samples were stored in MACS Tissue Storage Solution and immediately transferred to the laboratory. Tissues were dissociated using the Tumor Dissociation Kit, human, in combination with gentleMACS Octo Dissociator with heaters (all Miltenyi Biotec, Bergisch Gladbach, Germany) according to the manufacturer’s instructions. Cells were cryopreserved and thawed following the above protocol.

### 2.6. Flow Cytometry

Fc Beads (BD Biosciences, Franklin Lakes, NY, USA) and fluorescence minus one (FMO) were used to determine spillover values and to identify the positive and negative populations prior to the analysis of patients’ samples.

PBMCs at a density of 1 × 10^6^/tube were incubated with the 100-fold diluted APC-Cy7 anti-TREM2 antibody (clone 2B5; Novus Biologicals, Centennial, CO, USA) for 15 min at RT in the dark. Cells were washed with PBS using centrifugation (5 min at 1500× g) and were resuspended in 100 µL of 4% formalaldehyde (Cell Signaling Technology) for fixation. After 15 min, cells were washed with PBS (5 min at 1500× g). Cells were permeabilized by adding 0.5% Tween 20 (Thermo Fisher Scientific) for 15 min, followed by a washing step. Cells were blocked with human FcR blocking reagent (BD Biosciences) for 10 min. Next, cells were incubated at RT for 15 min in the dark with PE anti-human CD68 (clone Y1/82A; BioLegend, San Diego, CA, USA), BV421 anti-human CD163 (clone GHI/61; BioLegend), and Alexa Fluor 647 anti-human CD206 antibodies (clone 15-2; Biolegend). Cells were analyzed using BD FACSVerse Flow Cytometer (BD Biosciences). FlowJo software 10.70. (Treestar, Woodburn, OR, USA) was used to examine cells and analyze flow cytometry data.

### 2.7. Enzyme-Linked Immunosorbent Assay

The serum concentrations of sTREM2 were measured with a commercial ELISA kit following the instructions of the manufacturer (ab224881; Abcam). Optical density was measured using a microplate reader (Thermo Fisher Scientific) at a wavelength of 450 nm. Concentrations were calculated referring to the standard curve.

### 2.8. Statistical Analysis

Statistical analyses were performed using Statistical Package for the Social Sciences 27.0 (SPSS, Chicago, IL, USA) and GraphPad Prism 7.01 software.

The relationships among variables were analyzed using Spearman’s rank correlation test. The statistical comparisons between the two groups were evaluated using a *t*-test.

The one-way analysis of variance (ANOVA) followed by a multiple comparisons test was used for comparisons among more than two groups. A survival analysis was carried out using the Kaplan–Meier method. The Log-rank test was used to determine differences between the groups. Potential prognostic factors were analyzed using univariate and multivariate Cox regression.

A *p*-value < 0.05 was considered statistically significant.

## 3. Results

### 3.1. Patient Cohort for Tissue Microarrays

Immunohistochemical staining on TMAs was performed to evaluate the expression of TREM2 in the primary tumors and LNMs of OSCC patients. A total of 171 primary tumors were investigated. Of these patients, 100 (58.5%) were male, and 71 (41.5%) were female. The ages ranged from 28 to 88 years with a mean age of 65 years. Table S1 provides an overview of the clinicopathological characteristics of the patient cohort.

### 3.2. Correlation of TREM2 Expression in Primary Tumors with Clinicopathological Characteristics

IHC showed a mainly membranous and cytoplasmatic staining pattern of TREM2. TREM2^+^ cells were mostly found intratumorally.

**Figure 1:**
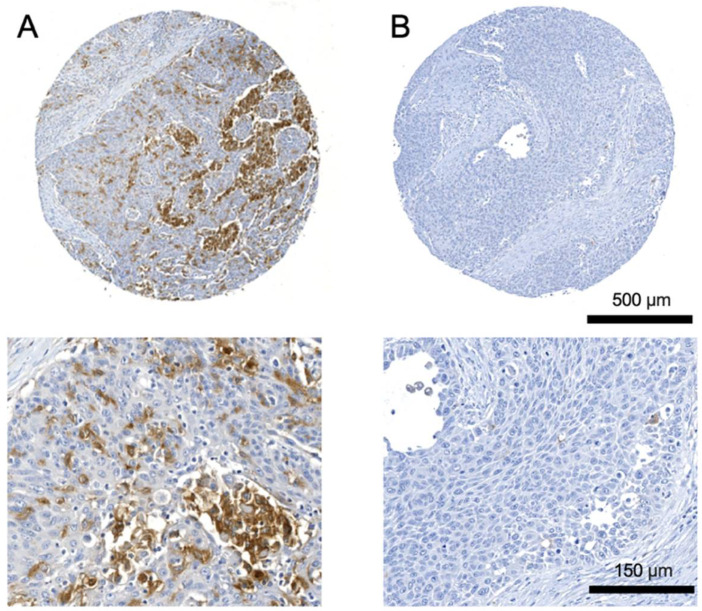
Representative images of tissue microarrays showing (**A**) strong and (**B**) low TREM2 expression in primary tumors of oral squamous cell carcinoma patients. Zoom images of selected regions from the cores are shown underneath.

High TREM2 expression was significantly correlated with female sex (Spearman’s rank correlation (SRC, *p* = 0.021)), high T classification (SRC, *p* = 0.03), high UICC stage, (SRC, *p* = 0.04), and high PD-L1 expression (SRC, *p* < 0.001). Moreover, tumor localization at the lower jaw, upper jaw, and soft palate was correlated with high TREM2 expression (SRC, *p* = 0.023). Patients’ clinicopathological characteristics according to TREM2 expression in primary tumor are shown in Table 1. Representative images of TMAs showing different staining intensities are shown in Figure 1.

### 3.3. Survival Analysis in Relation to TREM2 Expression

**Figure 2:**
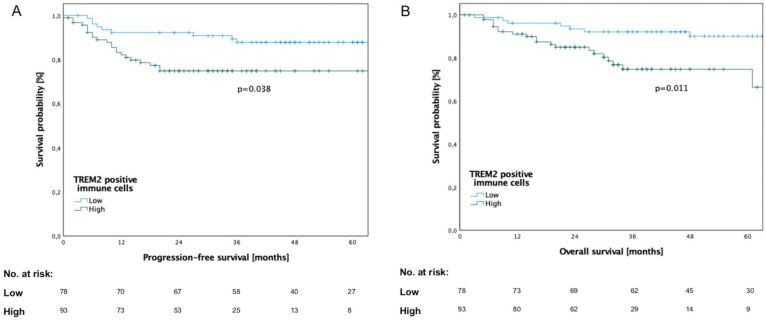
Kaplan–Meier curves of progression-free survival (PFS) and overall survival (OS) for 171 patients with OSCC according to TREM2 expression in primary tumor. (**A**) Patients with high TREM2 expression showed significantly inferior PFS (*p* = 0.038). (**B**) Patients with high TREM2 expression showed significantly inferior OS (*p* = 0.011).

The analysis revealed a significantly reduced progression-free (PFS; Log-rank test, *p* = 0.038) and overall survival (OS; Log-rank test, *p* = 0.011) for patients with high TREM2 expression (Figure 2). The mean time to death was 49.15 ± 17.61 months in the low-TREM2-expression group, whereas it was 31.13 ± 17.82 months in the high-TREM2-expression group.

To further assess the prognostic value of TREM2 expression and clinicopathological characteristics, univariate and multivariate Cox regression analyses were performed.

Univariate Cox regression revealed the UICC stage (*p* = 0.004) and the number of TREM2^+^ immune cells (*p* = 0.038) as significant prognostic factors regarding PFS (Table S2). Multivariate Cox regression confirmed the UICC stage (*p* = 0.005) and the number of TREM2^+^ immune cells (*p* = 0.044) as independent prognostic factors regarding PFS (Table S3). Similarly, the UICC stage (univariate, *p* = 0.004, multivariate, *p* = 0.005) and the number of TREM2^+^ immune cells (univariate, *p* = 0.038; multivariate, *p* = 0.044) were confirmed as prognostic factors in univariate and multivariate Cox regression regarding OS. The results of univariate and multivariate analyses are shown in Tables S2 and S3.

### 3.4. Expression of TREM2 in Lymph-Node Metastases

Next, immunohistochemical staining on TMAs was performed to evaluate the expression of TREM2 in the LNMs of 33 OSCC patients. Table S4 provides an overview of the clinicopathological characteristics of the patient cohort. Representative images of TMAs showing different staining intensities are exemplified in Figure S1. The abundance of TREM2^+^ cells was balanced between intra- and perimetastatic areas.

High TREM2 expression significantly correlated with female sex (SRC, *p* = 0.008), age > 75 years (SRC, *p* = 0.042), and high PD-L1 expression (SRC, *p* < 0.001). The results of the correlation analysis are shown in Table S5.

Patients with high TREM2 expression showed significantly inferior OS (*p* = 0.005). No significant differences in PFS were found between the low- and high-TREM2-expression groups (*p* = 0.105) (Figure S2).

Univariate Cox regression revealed the number of TREM2^+^ immune cells as a prognostic factor regarding overall survival (*p* = 0.005). In multivariate analysis, the number of TREM2^+^ immune cells was marginally significant (*p* = 0.05). The results of the univariate and multivariate analyses are shown in Tables S6 and S7.

No significant differences were observed between TREM2 expression in primary tumors and LNMs (*t*-test, *p* = 0.330). Further investigation showed that TREM2 expression was significantly upregulated in LNMs compared with primary tumors in men (*t*-test, *p* < 0.001), whereas it was similar in women (*t*-test, *p* = 0.669).

### 3.5. Patient Cohort for Flow Cytometric Analysis and Enzyme-Linked Immunosorbent Assay

The clinicopathological characteristics of the patient cohort for the flow cytometric analysis and enzyme-linked immunosorbent assay are shown in Table S8.

The mean age was 67 years in the OSCC group and 63 years in the HC group. There were no statistically significant differences in mean age (*t*-test, *p* = 0.987) nor gender (*t*-test, *p* = 0.522) between OSCC patients and HCs.

### 3.6. Expression of CD68, CD163, CD206, and TREM2 in PBMCs

**Figure 3:**
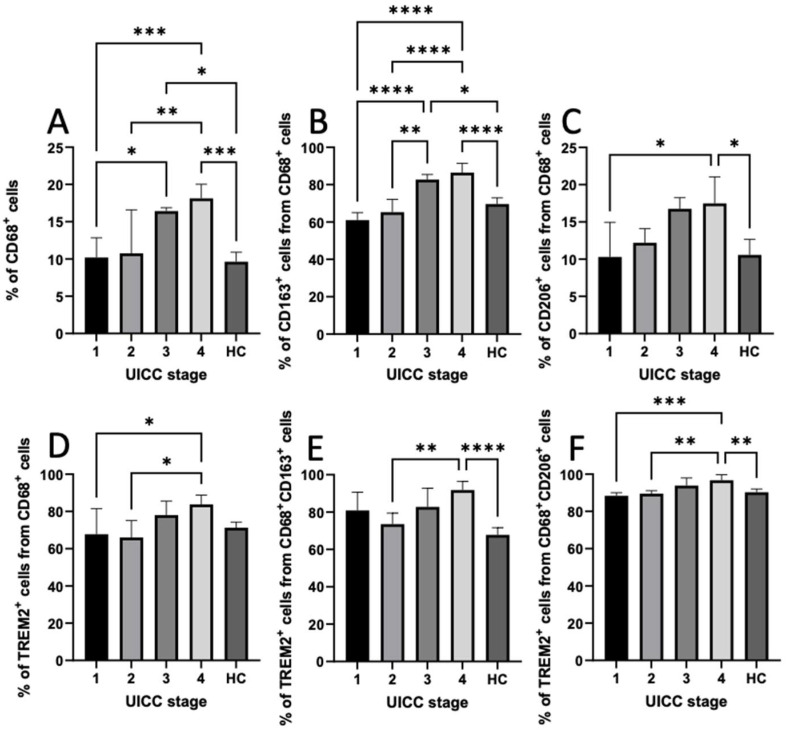
Quantification and comparison of different circulating macrophage subsets between oral squamous cell carcinoma patients and healthy control (HC) group depending on UICC stage. Graphs represent the percentages of (**A**) CD68^+^ cells, (**B**) CD163^+^ cells, (**C**) CD206^+^ cells gated on CD68^+^ cells, (**D**) TREM2^+^ cells gated on CD68^+^ cells, (**E**) TREM2^+^ cells gated on CD68^+^CD163^+^, and (**F**) TREM2^+^ cells gated on CD68^+^CD206^+^ cells. Statistical analyses were performed using the one-way ANOVA, followed by a multiple comparisons test. Values were expressed as means ± standard deviation. Asterisks represent relevant statistical differences between groups. * indicates *p*-value < 0.05, ** indicates *p*-value < 0.01, *** indicates *p*-value < 0.001, and **** indicates *p*-value < 0.0001.

We used flow cytometry to evaluate the percentage of circulating macrophages with regard to the expression of TREM2 in PBMCs. Figure S3 illustrates the gating strategy used to identify the macrophages. We found a significant correlation between CD68^+^, CD68^+^CD163^+^, and CD68^+^CD206^+^ cells and the UICC stage (SRC, *p* < 0.05). In addition, the results of the t-test revealed a significant increase in circulating CD68^+^, CD68^+^CD163^+^, and CD68^+^CD206^+^ macrophages in UICC stage IV compared with UICC stage I and HCs (ANOVA, *p* < 0.05). Moreover, we identified significantly higher proportions of TREM2^+^ cells among CD68^+^CD163^+^ and CD68^+^CD206^+^ macrophages in UICC stage IV compared with UICC stage I and HCs (ANOVA, *p* < 0.05). The percentage of TREM2^+^ cells among CD68^+^ cells was significantly higher in UICC stage IV than in stages I and II (ANOVA, *p* < 0.05; Figure 3). However, there were no significant differences regarding the percentage of TREM2^+^ cells among CD68^+^ cells with respect to HCs. However, a significant correlation was found between the age and the number of CD68^+^CD163^+^ cells (*p* = 0.049).

### 3.7. Distribution of TAMs in Intratumoral and Peritumoral Regions

To examine the localization of TAMs, the flow cytometric analyses of single-cell suspensions from tumor and healthy adjacent tissues were performed.

**Figure 4:**
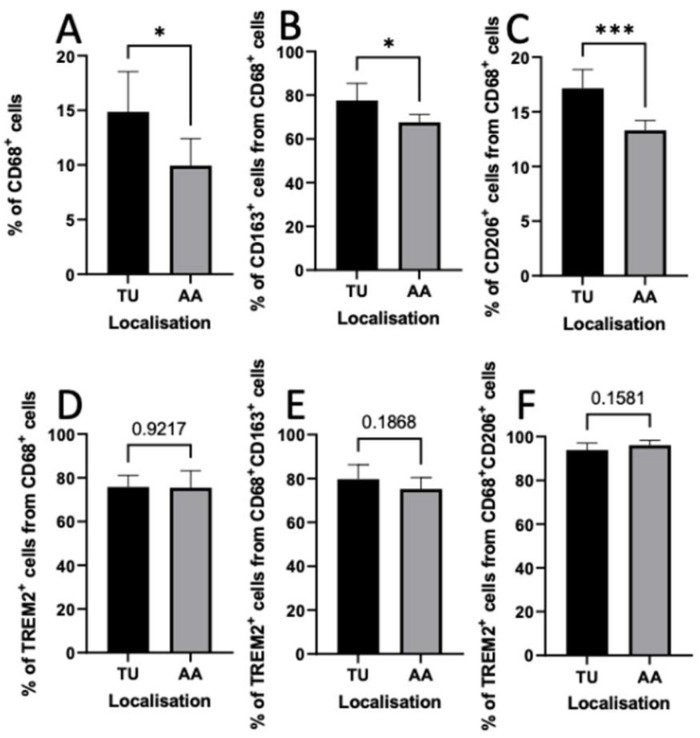
Quantification and comparison of different macrophage subsets between tumor tissue (TU) and adjacent area (AA) in oral squamous cell carcinoma patients. Graphs represent the percentages of (**A**) CD68^+^ cells, (**B**) CD163^+^ cells, (**C**) CD206^+^ cells gated on CD68^+^ cells, (**D**) TREM2^+^ cells gated on CD68^+^ cells, (**E**) TREM2^+^ cells gated on CD68^+^CD163^+^, and (**F**) TREM2^+^ cells gated on CD68^+^CD206^+^ cells. Values were expressed as means ± standard deviation. Asterisks represent relevant statistical differences between groups. * indicates *p*-value < 0.05 and *** indicates *p*-value < 0.001.

We identified higher proportions of CD68^+^, CD68^+^CD163^+^, and CD68^+^CD206^+^ TAMs in tumor tissue than in the adjacent area (t-test, *p* < 0.05). However, there were no significant differences regarding the TREM2 expression in TAMs in intratumoral and peritumoral regions (*t*-test, *p* > 0.05) (Figure 4).

### 3.8. Serum Levels of Soluble TREM2

The serum levels of sTREM2 were measured via ELISAs. The levels of sTREM2 were higher in advanced UICC stages (III/IV) than in lower UICC stages (I/II) and HCs. However, the differences did not reach significance (ANOVA, *p* = 0.401; Figure S4). We examined the association of sTREM2 levels with clinicopathological characteristics. As shown in Table S9, correlation with UICC stage (SRC, *p* = 0.059) was almost significant.

## 4. Discussion

Recently, TREM2 was identified as a potential therapeutic target in cancer therapy. Moreover, TREM2 was found on TAMs in the TME. Increasingly more evidence suggests that TAMs are a major source of resistance to therapy and thus limited prognosis. However, the significance of TREM2 remains unclear. To our knowledge, this is the first study regarding the role of TREM2 and its link to TAMs in OSCC.

In the present study, we investigated the association of TREM2 expression in immune cells in primary tumors and LNMs with clinicopathological characteristics and survival rate via IHC on TMAs. The object of the study was a large homogeneous cohort of OSCC patients with primarily surgical therapy, according to treatment guidelines. We observed that high TREM2 expression in primary tumors and LNMs was correlated with aggressive pathological characteristics and worse prognosis in OSCC patients.

However, previous results regarding the role of TREM2 in different malignancies are contradictory. Our results are similar to those obtained in gastric cancer [26] and renal cell carcinoma [27]. In contrast, the upregulation of TREM2 expression is considered to hamper tumor progression in hepatocellular carcinoma [28], colorectal cancer [29], and lung cancer [30].

Cheng et al. reported in their systematic pan-cancer analysis that the expression of TREM2 was elevated in HNSCC compared with noncancerous mucosa. However, no differences were evident in the survival rates between patients with high and low TREM2 expression, which might have been due to the fact that they performed a survival analysis based on expression at the gene level [31].

TREM2 expression was significantly upregulated in LNMs compared with primary tumors in men (*t*-test, *p* < 0.001), whereas it was similar in women (*t*-test, *p* = 0.669). Further investigations are needed to elucidate if there are gender-specific differences in the immune response and thus TREM2 expression by immune cells upon exposure to lymphatic tissue. Clinical investigations may show if there are gender-specific differences in the efficacy of anti-TREM2 immunotherapy in the stages of metastatic disease.

Yao et al. demonstrated that TREM2 was overexpressed in the PBMCs and TAMs of patients with lung cancer and tumor-bearing mice compared with HCs. Moreover, they observed a positive correlation between the level of TREM2 expression in pulmonary macrophages and the UICC stages as well as the degree of LNM [30]. In contrast to these results, we were able to show, using flow cytometry, that TREM2 expression was even significantly increased in PBMCs in UICC stage IV in OSCC patients compared with HCs (ANOVA, *p* < 0.05). However, TREM2 expression in OSCC patients in UICC stages I and II was similar to that in HCs.

TAMs become activated after infiltrating the TME and then exhibit two distinct subtypes. On the one hand, they exhibit the classically activated, pro-inflammatory (anti-tumoral) M1 phenotype. On the other hand, the alternatively activated, anti-inflammatory (pro-tumoral) M2 phenotype is expressed. Macrophage phenotypes are characterized by different surface markers: CD68 is a pan-macrophage marker; M1 macrophages typically exhibit CD11c, and the M2 phenotype highly expresses CD163, CD204, or CD206 [32,33,34]. It is widely accepted that the conventional M1/M2 classification has limitations in terms of explaining macrophage plasticity and that TAMs are not a homogenous subset of cells [35]. Therefore, we investigated the role of CD68^+^CD163^+^ and CD68^+^CD206^+^ TAMs and their link to TREM2 separately. Unlike previous studies using IHC, we applied flow cytometry to identify macrophages and TAM subsets and their link to TREM2 expression. We considered CD68 plus CD163 or CD206 for allocating macrophages towards M2 polarization, whereas most authors only consider single-staining with CD163, which might not be sufficient for allocating macrophages towards M2 polarization [36]. Moreover, CD163 is widely used to evaluate TAMs, whereas CD206 has rarely been investigated in the past.

Kumar et al. observed in their meta-analysis that an increase in TAMs was associated with a significant increase in the risk of high T classification and nodal positivity in HNSCC patients [37]. He at al. also identified that the intensity of the infiltration of CD68^+^ and CD163^+^ macrophages was positively correlated with lymph-node involvement and tumor size [38]. Suárez-Sánchez et al. discovered that higher infiltrations of CD68^+^ and CD163^+^ cells could be observed in larger tumors (T3, T4), advanced stages (III, IV), and moderate/poorly differentiated tumors, although differences did not attain statistical significance [36]. In contrast, Haque et al. did not find a significant association between the number of CD163^+^ and the clinicopathological characteristics of OSCC patients. On the contrary, they found that the number of CD206^+^cells was associated with UICC stage, T classification, and cervical-node metastasis (*p* < 0.05) [39]. The above-mentioned authors investigated the number of macrophages in tumor tissue, while we were able to show that the percentages of CD68^+^, CD68^+^CD163^+^, and CD68^+^CD206^+^ cells in PBMCs was significantly higher in advanced UICC stages than in limited stages and HCs (*p* < 0.05, ANOVA).

We investigated the density and tissue distribution of TREM2^+^ cells and TAMs by means of IHC and flow cytometry. Our results confirmed the previous findings that the expression of CD68 in tumor tissue was significantly higher than that in healthy adjacent tissue (ANOVA, *p* < 0.05) [40]. In addition, we discovered that the percentages of CD68^+^CD163^+^ and CD68^+^CD206^+^ cells in tumor tissue were significantly higher than in the adjacent healthy tissue (ANOVA, *p* < 0.05). We observed a higher number of TREM2^+^ immune cells in the intratumoral region than in the peritumoral region using IHC. However, we found a similar percentage of TREM2^+^ TAMs in the flow cytometric analyses of tumor tissue and healthy adjacent tissue. This might have been due to the fact that TREM2 is also expressed in dendritic cells [41].

While sTREM2 levels have been extensively studied in cognitive decline, few studies have investigated sTREM2 levels in cancer. Decreased levels of sTREM2 were detected in patients with dementia, while increased levels of sTREM2 were detected in the cerebrospinal fluid of inflammatory neurological diseases, e.g., multiple sclerosis [42]. However, up to now, the serum levels of sTREM2 in patients with malignancies have only been investigated in high-grade-glioma patients. Therefore, we conducted an ELISA for measuring the serum levels of sTREM2. In contrast to high-grade-glioma patients, OSCC patients showed higher levels of sTREM2 in advanced UICC stages [43]. However, this trend did not reach statistical significance (ANOVA, *p* = 0.401, Figure S4). Nevertheless, levels of sTREM2 were almost significantly correlated with UICC stages (SRC, *p* = 0.059). Therefore, the suitability of sTREM2 as a biomarker in OSCC patients should be investigated in a larger patient cohort.

This study had some limitations that should be taken into consideration. The small number of patients might have given the flow cytometric analysis lower power. Nevertheless, our findings were consistent with those obtained by others, supporting and extending the previous view. Unlike previous studies using IHC, we used flow cytometry for identifying macrophages and TAM subsets and their link to TREM2 expression. Flow cytometry enabled us to determine several markers on cells. However, since one needs a certain amount of tissue for dissociation that is not examined by a pathologist, using flow cytometry has the disadvantage of only being able to investigate T2 and larger tumors.

## 5. Conclusions

Overall, we found that high TREM2 expression played a critical role in OSCC patients and was associated with an advanced UICC stage and reduced survival rates. The significant upregulation of TREM2 in LNMs compared with primary tumors in men may indicate gender-specific differences in the immune response. Our findings implicated that TREM2 could serve as an effective target for prognostic and therapeutic approaches in OSCC patients. Anti-TREM2 immunotherapy might greatly improve patients’ prognosis. High levels of sTREM2 were associated with advanced UICC stages; however, the correlation did not reach significance. The suitability of sTREM2 as a biomarker in OSCC patients should be investigated in a larger patient cohort.

## Figures and Tables

**Table 1:** Correlation of TREM2 expression with clinicopathological characteristics of oral squamous cell carcinoma patients.

Characteristic		Low TREM2 Expression in Immune Cells (%)	High TREM2 Expression in Immune Cells (%)	*p*-Value
Sex	Men	53 (53)	47 (47)	0.021 *
	Women	25 (35.2)	46 (64.8)	
Age	≤ 75	66 (48.9)	69 (51.1)	0.097
	> 75	12 (33.3)	24 (66.7)	
T classification	1	32 (54.2)	27 (45.8)	0.030 *
	2	28 (48.3)	30 (51.7)	
	3	2 (28.6)	5 (71.4)	
	4	16 (34)	31 (66)	
N classification	0	60 (50.4)	59 (49.6)	0.05
	1	8 (42.1)	11 (57.9)	
	2b	5 (26.3)	14 (73.7)	
	2c	5 (35.7)	9 (64.3)	
UICC stage	I	28 (53.8)	24 (46.2)	0.04 *
	II	19 (50)	19 (50)	
	III	9 (50)	9 (50)	
	IV	22 (34.9)	41 (65.1)	
Recurrence	No	67 (48.5)	71 (51.5)	0.116
	Yes	11 (33.3)	22 (66.7)	
Differentiation grade	1	13 (86.7)	2 (13.3)	0.102
	2	48 (41)	69 (59)	
	3	15 (44.1)	19 (55.9)	
	Missing	2 (40)	3 (60)	
Localization	Floor of the mouth	26 (55.3)	21 (44.7)	0.023 *
	Tongue	23 (54.7)	19 (45.3)	
	Lower jaw	20 (38.5)	32 (61.5)	
	Upper jaw	1 (33.3)	2 (66.7)	
	Lower lip	0 (0)	1 (100)	
	Soft palate	2 (18.2)	9 (81.8)	
	Buccal plane	6 (50)	6 (50)	
	Missing	0 (0)	3 (100)	
PD-L1	Negative	33 (73.3)	12 (26.7)	<0.001 *
	Positive	36 (33.3)	69 (63.9)	
	Missing	9 (34.3)	12 (65.7)	
PD-L2	Negative	15 (62.5)	9 (37.5)	0.088
	Positive	53 (43.4)	69 (56.6)	
	Missing	10 (40)	15 (60)	
p16	Negative	43 (47.7)	47 (52.3)	0.7
	Positive	24 (44.4)	30 (55.6)	
	Missing	11 (40.7)	16 (59.3)	

Abbreviations: DNA = desoxyribonucleic acid; PD-L1/2 = Programmed cell death ligand 1/2; UICC = Union Internationale Contre le Cancer. * indicates *p*-value < 0.05.

## Data Availability

The data that support the findings of this study are available from the corresponding author upon reasonable request.

## Supplementary Materials

The following supporting information can be downloaded at: https://www.mdpi.com/article/10.3390/cancers14194635/s1, Table S1: Clinicopathological characteristics of the investigated tissue microarray cohort, Table S2: Univariate analysis of clinicopathological characteristics and TREM2 expression in primary tumors of oral squamous cell carcinoma patients, Table S3: Multivariate analysis of clinicopathological characteristics and TREM2 expression in primary tumors of oral squamous cell carcinoma patients, Table S4: Clinicopathological characteristics of the investigated tissue microarray cohort with lymph-node metastases, Table S5: Correlation of TREM2 expression in lymph-node metastases with clinicopathological characteristics of oral squamous cell carcinoma patients, Table S6: Univariate analysis of clinicopathological characteristics and TREM2 expression in lymph-node metastases of oral squamous cell carcinoma patients, Table S7: Multivariate analysis of clinicopathological characteristics and TREM2 expression in lymph-node metastases of oral squamous cell carcinoma patients, Table S8: Descriptive data regarding demographic and clinicopathological characteristics of the prospective cohort of oral squamous cell carcinoma patients and healthy controls, Table S9: Correlation analysis of levels of soluble TREM2 and clinicopathological characteristics of oral squamous cell carcinoma patients, Figure S1: Representative images of tissue microarrays. Figure S2: Kaplan–Meier curves of progression-free survival (PFS) and overall survival (OS) for 33 patients with OSCC according to TREM2 expression in lymph-node metastases, Figure S3: Flow cytometric analysis of CD68, CD163, CD206, and TREM2 expression in macrophages. Figure S4: Scatter plot depicting the relationship between the amount of soluble TREM2 in the serum of oral squamous cell carcinoma patients and healthy controls (HCs), and the UICC stage.
