# Supplementary material for: TREM2 Is Associated with Advanced Stages and Inferior Prognosis in Oral Squamous Cell Carcinoma
Source: Cancers (Basel). 2022 Sep 24;14(19):4635. doi: 10.3390/cancers14194635 (PMC9561992; doi:10.3390/cancers14194635)
Supplement: Supplementary file 1 [file cancers-14-04635-s001.zip › cancers-1860752-supplementary.pdf]

Supplementary Material: TREM2 Is Associated with Advanced Stages and Inferior Prognosis in Oral Squamous Cell Carcinoma

Ann-Kristin Struckmeier *, Anne Radermacher, Michael Fehrenz, Dalia Alansary, Philipp Wartenberg, Mathias Wagner, Anja Scheller, Jochen Hess, Julius Moratin, Christian Freudlsperger, Jürgen Hoffmann, Lorenz Thurner, Klaus Roemer, Kolja Freier and Dominik Horn

Table S1. Clinicopathological characteristics of the investigated tissue microarray cohort.

Characteristics	Number of patients (%)
**Sex**	
**Men**	100 (58.5)
**Women**	71 (41.5)
**Age**	
**≤ 75 y**	135 (78.9)
**> 75 y**	36 (21.1)
**T classification**	
**1**	59 (34.5)
**2**	58 (33.9)
**3**	7 (4.1)
**4**	47 (27.5)
**N classification**	
**0**	119 (69.6)
**1**	19 (11.1)
**2b**	19 (11.1)
**2c**	14 (8.2)
**M classification**	
**0**	171 (100)
**1**	0 (0)
**UICC stage**	
**I**	52 (30.4)
**II**	38 (22.2)
**III**	18 (10.5)
**IV**	63 (36.8)
**Differentiation grade**	
**1**	15 (8.8)
**2**	117 (68.4)
**3**	37 (21.6)
**Missing**	2 (1.2)
**Location**	
**Floor of the mouth**	47 (26.9)
**Tongue**	43 (24.6)
**Lower jaw**	55 (31.4)
**Upper jaw**	3 (1.7)
**Soft palate**	11 (6.3)
**Buccal plane**	12 (6.9)
**Missing**	2 (1.1)
**Recurrence**	
**Yes**	33 (19.3)
**No**	138 (80.7)
**P16**	
**Positive**	54 (31.6)
**Negative**	90 (52.6)

**Missing**	27 (15.8)
**HPV-DNA**	
**Negative**	144 (84.3)
**Missing**	27 (15.7)

Abbreviations: DNA = desoxyribonucleic acid, HPV = human papillomavirus, UICC = Union Internationale Contre le Cancer.

Table S2. Univariate analysis of clinicopathological characteristics and TREM2 expression in primary tumours of oral squamous cell carcinoma patients.

Characteristics		Overall survival		Progression-free survival	
		**HR (95% CI)**	**P value**	**HR (95% CI)**	**P value**
**Sex**	Men vs. Women	1.16 (0.548-2.453)	0.698	0.809 (0.398-1.646)	0.557
Age	≤ 75 years vs. > 75 years	1.487 (0.627-3.526)	0.364	1.112 (0.482-2.569)	0.802
UICC stage	I/II vs. III/IV	2.929 (1.316-6.516)	0.006*	2.826 (1.135-5.916)	0.004*
TREM2$^+$ immune cells	Low vs. high	2.816 (1.224-6.478)	0.011*	2.126 (1.023-4.418)	0.038*

Statistically significant differences between groups were determined by Cox proportional hazard model. Asterisk indicates p value <0.05.

Abbreviations: CI = confidence interval, HR = hazard ratio.

Table S3. Multivariate analysis of clinicopathological characteristics and TREM2 expression in primary tumours of oral squamous cell carcinoma patients.

Characteristics		Overall survival		Progression-free survival	
		**HR (95% CI)**	**P value**	**HR (95% CI)**	**P value**
**Sex**	Men vs. women	1.002 (0.467-2.152)	0.996	0.618 (0.298-1.28)	0.195
Age	≤ 75 years vs. > 75 years	1.328 (0.55-3.21)	0.528	1.027 (0.438-2.405)	0.951
UICC stage	I/II vs. III/IV	2.885 (1.282-6.493)	0.01*	2.876 (1.367-6.05)	0.005*
TREM2$^+$ immune cells	Low vs. high	2.548 (1.089-5.964)	0.031*	2.17 (1.021-4.613)	0.044*

Statistically significant differences between groups were determined by Cox proportional hazard model. Asterisk indicates p value <0.05.

Abbreviations: CI = confidence interval, HR = hazard ratio.

Table S4. Clinicopathological characteristics of the investigated tissue microarray cohort with lymph node metastases.

Characteristics	Number of cases (%)
**Sex**	
**Men**	25 (75.8)
**Women**	8 (24.2)
**Age**	
**≤ 75 years**	27 (81.8)
**> 75 years**	6 (18.2)
**T classification**	
**1**	5 (15.2)
**2**	11 (33.3)
**3**	4 (12.1)
**4**	13 (39.4)
**N classification**	

1	12 (36)
2b	15 (45.5)
2c	6 (18.2)
UICC stage	
III	8 (24.2)
IV	25 (75.8)
Differentiation grade	
2	25 (75.8)
3	7 (21.2)
Missing	1 (3)
Localisation	
Floor of the mouth	5 (15.2)
Tongue	11 (33.3)
Lower jaw	10 (30.3)
Soft palate	2 (6.1)
Buccal plane	4 (12.1)
Missing	1 (3)
Recurrence	
Yes	11 (33.3)
No	22 (66.7)
p16	
Negative	10 (30.3)
Positive	9 (27.3)
Missing	14 (42.4)
HPV-DNA	
Negative	19 (57.5)
Missing	14 (42.4)

Abbreviations: DNA = desoxyribonucleic acid, HPV = human papillomavirus, UICC = Union Internationale Contre le Cancer.

Table S5. Correlation of TREM2 expression in lymph node metastases with clinicopathological characteristics of oral squamous cell carcinoma patients.

Characteristics		Low TREM2 expression in immune cells (%)	High TREM2 expression in immune cells (%)	P value
Sex	Men	6 (24)	19 (76)	0.008*
	Women	6 (75)	2 (25)	
Age	≤ 75 years	12 (44.4)	15 (55.6)	0.042*
	> 75 years	0 (0)	6 (100)	
T classification	1	4 (80)	1 (20)	0.054
	2	4 (36.4)	7 (63.6)	
	3	1 (25)	3 (75)	
	4	3 (23.1)	10 (76.9)	
N classification	1	5 (45.5)	6 (54.5)	0.465
	2b	6 (40)	9 (60)	
	2c	1 (14.3)	6 (85.7)	
UICC stage	III	5 (62.5)	3 (37.5)	0.082
	IV	7 (28)	18 (72)	
Recurrence	No	9 (40.9)	13 (59.1)	0.386
	Yes	3 (27.3)	8 (72.7)	
Differentiation grade	2	9 (36)	16 (64)	0.75
	3	3 (42.9)	4 (57.1)	
	Missing	0 (0)	1 (100)	
Localisation	Floor of the mouth	1 (20)	4 (80)	0.5
	Tongue	7 (63.6)	4 (36.4)	
	Lower jaw	2 (20)	8 (80)	

	Soft palate	1 (50)	1 (50)	
	Buccal plane	1 (25)	3 (75)	
	Missing	0 (0)	1 (100)	
PD-L1	Negative	2 (100)	0 (0)	0.022*
	Positive	4 (22.2)	14 (77.8)	
	Missing	6 (46.2)	7 (53.8)	
PD-L2	Negative	1 (100)	0 (0)	0.13
	Positive	5 (26.3)	14 (73.7)	
	Missing	6 (46.2)	7 (53.8)	
p16	Negative	4 (40)	6 (60)	0.434
	Positive	2 (22.2)	7 (77.8)	
	Missing	6 (42.9)	8 (57.1)	

Asterisk indicates p value <0.05.

Abbreviations: DNA = desoxyribonucleic acid, PD-L1/2 = Programmed cell death ligand 1/2, UICC = Union Internationale Contre le Cancer.

Table S6. Univariate analysis of clinicopathological characteristics and TREM2 expression in lymph node metastases of oral squamous cell carcinoma patients.

Characteristics		Overall survival		Progression-free survival	
		HR (95% CI)	P value	HR (95% CI)	P value
**Sex**	Men vs. women	0.684 (0.193-2.429)	0.551	1.01 (0.325-3.137)	0.986
Age	≤ 75 years vs. > 75 years	4.196 (1.197-14.706)	0.104	3.642 (1.173-11.36)	0.105
UICC stage	III vs. IV	2.797 (0.627-12.474)	0.155	2.942 (0.665-13.02)	0.128
TREM2$^+$ immune cells	Low vs. high	6.713 (1.475-30.554)	0.005*	2.44 (0.782-7.61)	0.105

Statistically significant differences between groups were determined by Cox proportional hazard model. Asterisk indicates p value <0.05.

Abbreviations: CI = confidence interval, HR = hazard ratio.

Table S7. Multivariate analysis of clinicopathological characteristics and TREM2 expression in lymph node metastases of oral squamous cell carcinoma patients.

Characteristics		Overall survival		Progression-free survival	
		HR (95% CI)	P value	HR (95% CI)	P value
**Sex**	Men vs. women	0.73 (0.186-2.868)	0.652	1.356 (0.42-4.379)	0.611
Age	≤ 75 years vs. > 75 years	2.65 (0.681-10.304)	0.16	2.492 (0.731-8.489)	0.144
UICC stage	III vs. IV	1.762 (0.382-8.126)	0.468	2.123 (0.44-10.25)	0.349
TREM2$^+$ immune cells	Low vs. high	4.904 (1.003-23.987)	0.05	1.696 (0.464-6.201)	0.424

Statistically significant differences between groups were determined by Cox proportional hazard model.

Abbreviations: CI = confidence interval, HR = hazard ratio.

Table S8. Descriptive data regarding demographic and clinicopathological characteristics of the prospective cohort of oral squamous cell carcinoma patients and healthy controls.

Characteristics	Number of OSCC patients (%)	Number of patients in healthy control group (%)
**Sex**		
**Men**	9 (47.4)	2 (50)
**Women**	10 (52.6)	2 (50)
**Age**		
**≤75 y**	14 (73.7)	3 (75)
**>75 y**	5 (26.3)	1 (25)
**T classification**		
**T1**	4 (21.1)	
**T2**	4 (21.2)	
**T3**	3 (15.8)	
**T4**	8 (41.1)	
**N classification**		
**0**	14 (73.7)	
**1**	1 (5.3)	
**2a**	1 (5.3)	
**2b**	2 (10.5)	
**3b**	1 (5.3)	
**M classification**		
**0**	19 (100)	
**1**	0 (0)	
**UICC stage**		
**I**	4 (21.1)	
**II**	3 (15.8)	
**III**	3 (15.8)	
**IV**	9 (47.4)	
**Differentiation grade**		
**1**	2 (10.5)	
**2**	6 (31.6)	
**3**	8 (42.1)	
**Missing**	3 (15.8)	
**p16**		
**Negative**	19 (100)	
**HPV-DNA**		
**Negative**	19 (100)	

Abbreviation: DNA = desoxyribonucleic acid, HPV = human papillomavirus, UICC = Union Internationale Contre le Cancer.

Table S9. Correlation analysis of levels of soluble TREM2 and clinicopathological characteristics of oral squamous cell carcinoma patients.

Characteristics	Spearman's rho	P value
**Sex**	0.192	0.430
**Age**	-0.327	0.171
**T classification**	0.410	0.081
**N classification (N0/N+)**	0.405	0.085
**UICC stage**	0.441	0.059

Abbreviation: UICC = Union Internationale Contre le Cancer.

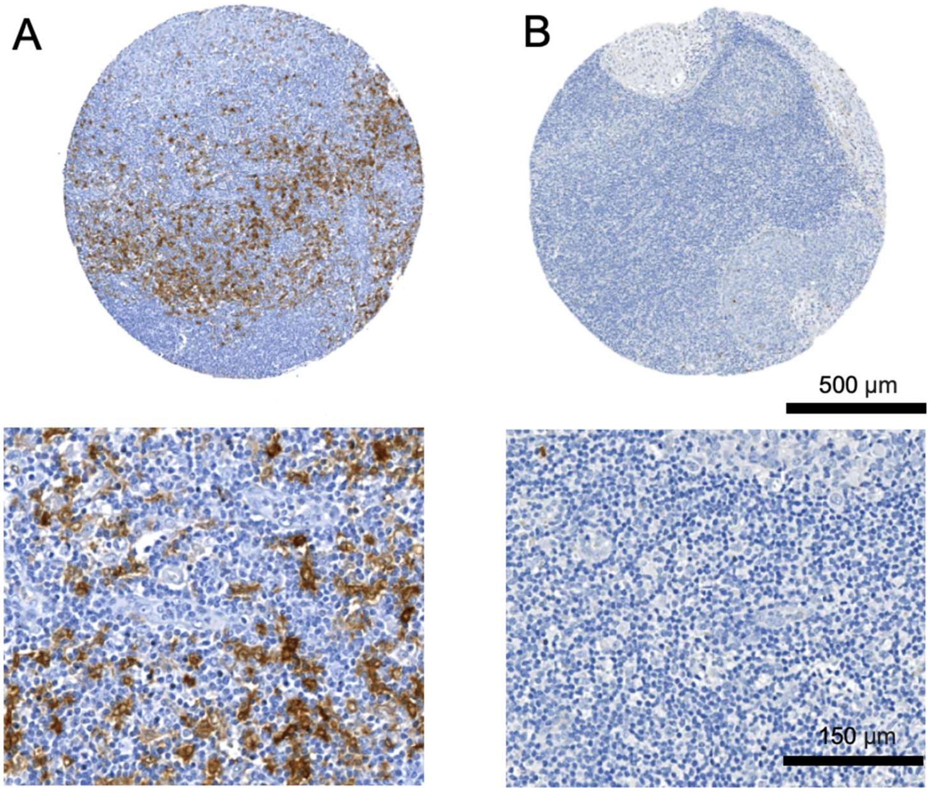

Figure S1. Representative images of tissue microarrays showing **(A)** strong and **(B)** low TREM2 expression in lymph node metastases of oral squamous cell carcinoma patients. Zoom images of selected region from the cores are shown below.

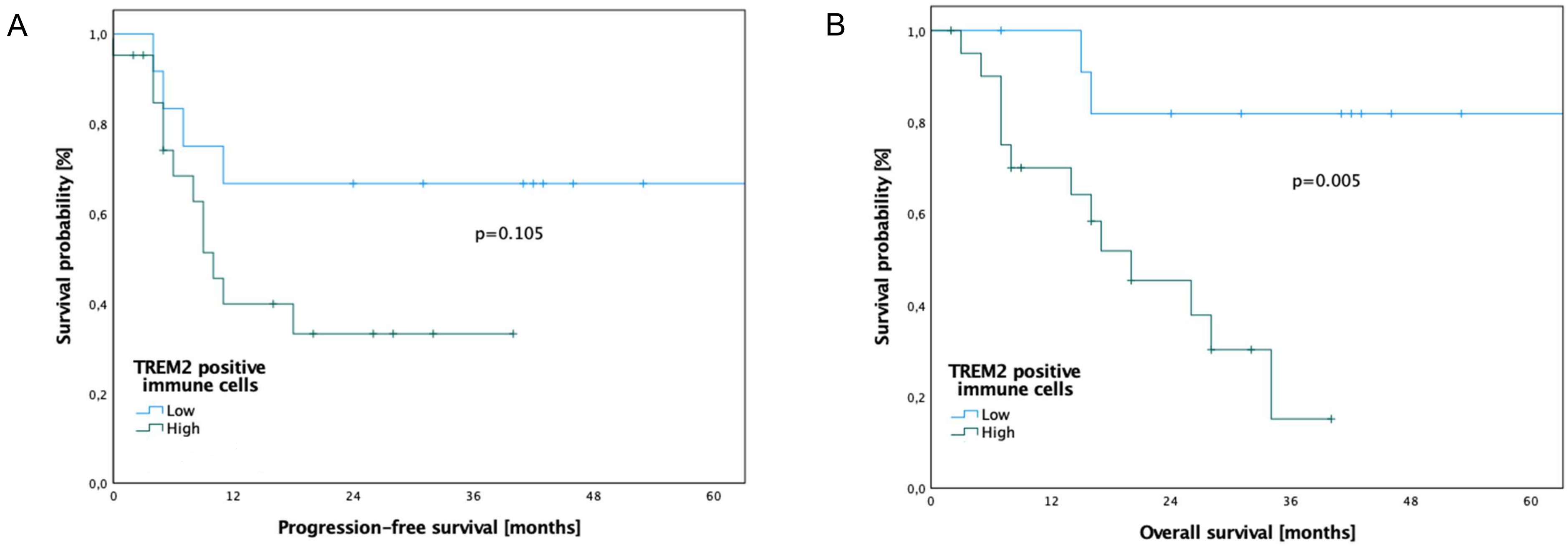

No. at risk:

**Low**	12	8	8	6	2	1
**High**	21	7	4	1	0	0

No. at risk:

**Low**	12	10	9	7	3	2
**High**	21	12	6	1	0	0

Figure S2. Kaplan-Meier curves of progression-free survival (PFS) and overall survival (OS) for 33 patients with OSCC according to TREM2 expression in lymph node metastases. **(A)** No significant difference in PFS was noted between low and high TREM2 expression groups (p=0.105). **(B)** Patients with high TREM2 expression showed significantly inferior OS (p=0.005).

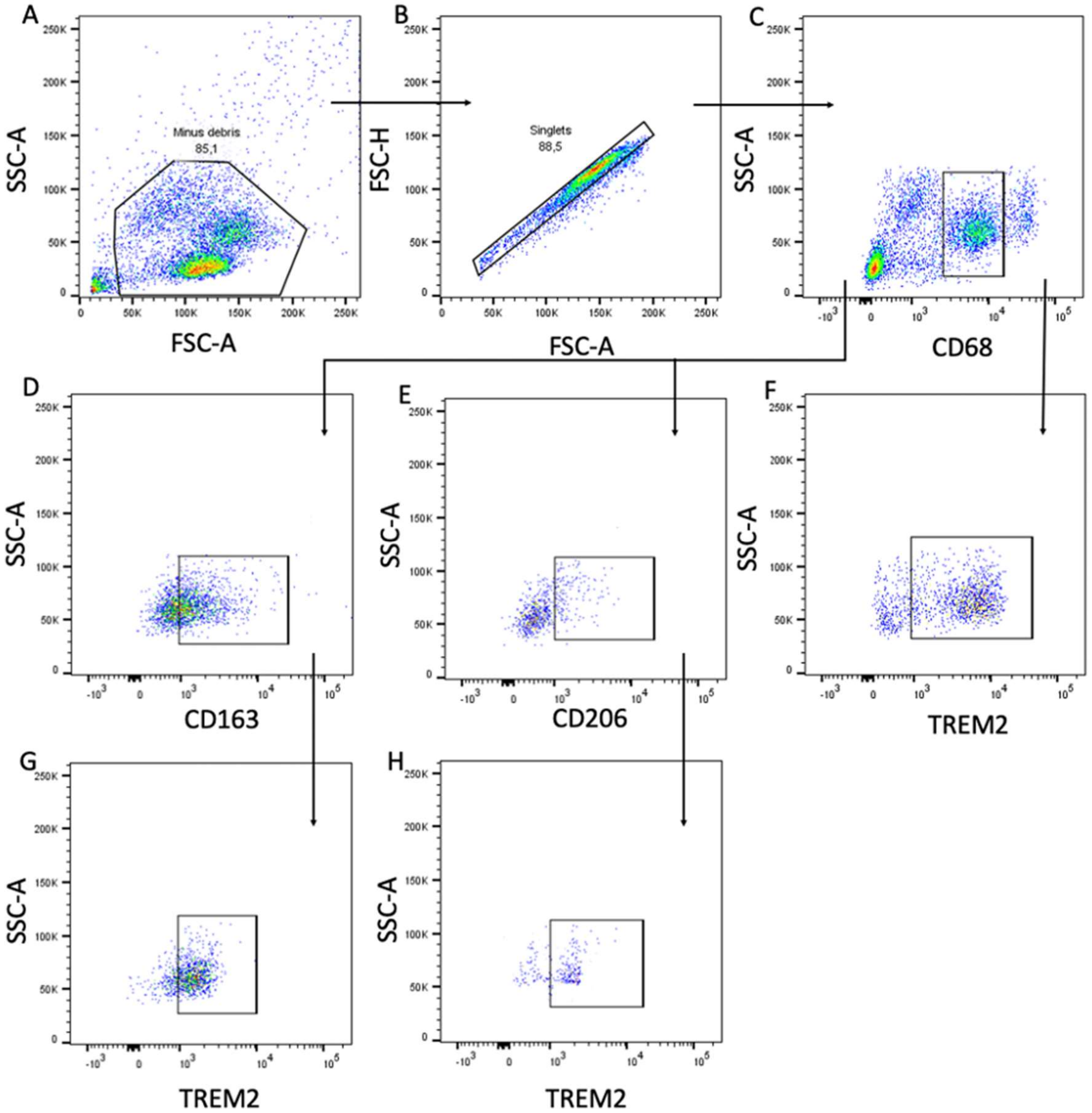

Figure S3. Flow cytometric analysis of CD68, CD163, CD206 and TREM2 expression on macrophages. **(A)** SSC-A and FSC-A were used as parameters to exclude cellular debris from all recorded events. **(B)** Single cells were gated using Figure 68. cells were defined as macrophages. From the CD68 gate, the expression of **(D)** CD163 and **(E)** CD206 was assed. The percentage of TREM2$^+$ cells was investigated from the **(F)** CD68$^+$ cells, **(G)** CD68$^+$CD163$^+$ cells, and **(H)** CD68$^+$CD206$^+$ cells.

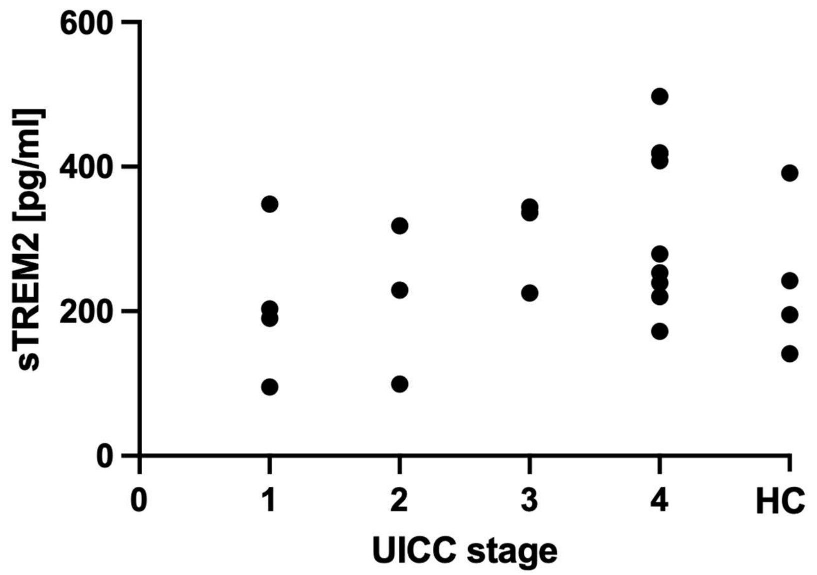

Figure S4. Scatter plot depecting the relationship between the amount of soluble TREM2 in serum of oral sqamous cell carcinoma patients and healthy controls (HCs) and the UICC stage.
